# Neonatal outcomes in obese mothers: a population-based analysis

**DOI:** 10.1186/1471-2393-13-36

**Published:** 2013-02-11

**Authors:** Anne-Frederique Minsart, Pierre Buekens, Myriam De Spiegelaere, Yvon Englert

**Affiliations:** 1 Perinatal Epidemiology Center ‘CEpiP’ School of Public Health, University Hospital Erasme and Faculty of Medicine, Université Libre de Bruxelles, Brussels, 1070, Belgium; 2 Department of Obstetrics and Gynecology and Research Laboratory for Human Reproduction, University Hospital Erasme and Faculty of Medicine, Université Libre de Bruxelles, Brussels, 1070, Belgium; 3 School of Public Health and Tropical Medicine, Tulane University, New Orleans, LA, USA; 4 Brussels-Capital Health and Social Observatory, Brussels, Belgium; 5 Service de Gynécologie-Obstétrique, Hôpital Universitaire Erasme, Université Libre de Bruxelles, Route de Lennik, 808, Brussels, 1070, Belgium

**Keywords:** Obesity (MeSH), Intensive care, Neonatal (MeSH), Apgar score (MeSH), Perinatal mortality (MeSH), Obstetric delivery (MeSH), Obstetric labor (MeSH)

## Abstract

**Background:**

If it is well known that obesity increases morbidity for both mother and fetus and is associated with a variety of adverse reproductive outcomes, then few studies have assessed the relation between obesity and neonatal outcomes. This is the aim of the present study after taking into account type of labor and delivery, as well as social, medical and hospital characteristics in a population-based analysis.

**Methods:**

This study used 2009 data from the Belgian birth register data pertaining to the regions of Brussels and Wallonia and included 38,675 consecutive births. Odds ratio and 95% confidence intervals for admission to neonatal intensive care unit, Apgar score, and perinatal mortality were calculated by logistic regression analyses adjusting for medical, social and hospital characteristics using obesity as the primary independent variable. The impact of analyzing all delivery sites together was tested using mixed-effect analyses.

**Results:**

The adjusted odds ratio for neonatal intensive care unit admission was higher for obese mothers by 38% compared to non-obese mothers (95% confidence interval (CI): 1.22-1.56), and by 45% (CI: 1.21-1.73) and 34% (CI: 1.10-1.63) after spontaneous and induced labour respectively. The adjusted odds ratio was 1.18 (CI: 0.86-1.63) after caesarean section. The adjusted odds ratio for 1 minute Apgar score inferior to 7 was higher for obese mothers by 31% compared to non-obese mothers (CI: 1.15-1.49) and by 26% (CI: 1.04-1.52) and 38% (CI: 1.12-1.69) after spontaneous and induced labour respectively. The adjusted odds ratio was 1.50 (CI: 0.96-2.36) after caesarean section. The adjusted odds ratio for perinatal mortality was 1.36 (CI: 0.75-2.45) for obese mothers compared to non-obese mothers.

**Conclusions:**

Neonatal admission to intensive care and low Apgar scores were more likely to occur in infants from obese mothers, both after spontaneous and induced labor.

## Background

The average body mass index (BMI) is increasing among all age categories, and women enter pregnancy at higher weights. It is well known that obesity increases morbidity for both mother and fetus, and is associated with a variety of adverse reproductive outcomes [1-4]. Diabetes, hypertensive disorders, fetal deaths, macrosomia, postdate pregnancies, cesarean sections have all been associated with maternal obesity yet the exact mechanisms have not been identified [1-4].

Few studies have assessed the relation between obesity and neonatal complications, and most studies reporting more admissions to neonatal intensive care unit and low Apgar scores in infants from obese women did not adjust for confounding factors [4,5]. Perinatal outcome was mostly assessed in all pregnancies taken as a whole with few distinctions between induced or spontaneous labor, and it has not yet been well explored if infant outcomes in obese mothers could be influenced by these factors [6,7]. The aim of the present study was to evaluate the association between obesity and neonatal outcomes such as admission to neonatal intensive care unit, Apgar score, and perinatal mortality when taking into account type of labor and delivery, as well as social, medical, and hospital characteristics in a population-based analysis.

## Methods

This is a population-based study using birth certificates from the birth registry of 2009. Data pertain to all births in two of the three Belgian regions excluding Flanders. The register is anonymous and publically available and accessible with the permission of the Brussels Health Observatory and the Health Department of the French Community of Belgium.

### Databases

The birth registry legally includes birth certificates of all live births and stillbirths from 500 grams or 22 weeks’ gestation. The registry also includes births to women staying in Belgium illegally and asylum seekers. In total, 47,344 consecutive births were considered for the analyses. Prepregnancy weight was registered at the first prenatal consultation ≤12 weeks or based on self-reported weight if the first consultation was held >12 weeks [2]. Obesity status was defined as obese (BMI > =30.0 kg/m^2^) or non –obese (BMI between 18.5 and 29.9 kg/m^2^) according to the World Health Organization’s definition [2]. Women <18 years were classified according to specific obesity cut-off points developed by Cole et al [8]. Data regarding body mass index were missing in 12.8% of births. Underweight mothers (n = 2602, 6.3%), were not included in the non-obese group as they carry specific risks [1] and 38,675 births were thus finally included in the present analysis. Data regarding neonatal intensive care unit admission and 1 minute Apgar score were missing in 4.4% and 1.1% of births respectively. Data regarding other factors were missing in ≤1% of births, except education: 14.9%. Elective cesarean section (CS) and induction of labor were defined as procedures which had been carried out before the onset of labor. Mothers’ origin was defined based on their nationality at birth. Macrosomia was defined by birth weight ≥4000 g. Weight gain was expressed as superior or inferior to the Institute of Medicine recommendations upper limits [9]. Perinatal mortality included stillbirths and neonatal mortality within the first week of life.

### Analysis

We first calculated the perinatal characteristics of obese and non-obese women. Differences in percentages between groups were compared by chi-square analyses.

Next, we conducted bivariate and multivariate analyses using successively admission to neonatal intensive care unit within 24 hours (NICU), 1 minute Apgar score inferior to 7, and perinatal mortality as the dependent variable. Obesity status was used as the primary independent variable. All potential confounding variables were categorical. Several multivariate logistic regression models were built: first in an explicative view, by including maternal characteristics: age ≥35 years, primiparous, weight gain and maternal height; second, by adding medical characteristic: hypertension, diabetes, macrosomia, gestational age, multiple birth to the first model; and third, by adding socio-economic characteristics to the second model: employment status, education, cohabiting status and maternal origin. We tested the impact of analyzing all delivery sites (50 maternity units in total) together by repeating the multivariate regression analyses with mixed-effects logistic regression models. The use of such regression modelling accounts for unmeasured factors at the hospital level. We treated the hospital effect as the random effect and the remaining factors as fixed effects.

The same analyses were repeated according to type of labor (induction of labor, elective CS or spontaneous labor).

To validate our results we also repeated analyses in pregnancies that ended up at 39 and 40 weeks of pregnancy to preclude an effect of early induction of labor or elective CS before 39 weeks and to preclude an effect of postdate pregnancies [4,7,10].

Adjusted odds ratio (OR) and 95% confidence interval (95%CI) were derived from the model and likelihood-ratio test *p*-values are presented in the result tables. Goodness of fit for the final model was evaluated with Hosmer-Lemeshow test. Models were tested for the presence of interactions and the hypothesis of non-correlation among errors was tested using postestimation commands including residuals and predicted values. Statistical calculations were undertaken using the STATA software (version 10.0, College Station, Texas, USA).

## Results

Table 1 shows maternal and pregnancy characteristics. Obese women accounted for 12.6% of births.

**Table 1:** Population characteristics

**Background characteristics**		**Non-obese**	**Obese**
		**(N = 33,818)**	**(N = 4857)**
		%	%
**Age ≥ 35 years**		19.1	23.1
**Multiparity**		53.8	64.3
**Height (cm)**	*<155*	4.4	6.5
	*155-179*	94.0	92.2
	≥*180*	1.6	1.2
**High weight gain**		33.6	50.3
**Hypertension**		3.8	14.4
**Diabetes**		4.3	11.2
**Nationality**	*Belgian*	56.2	56.9
	*Sub-Saharan Africa*	6.1	9.3
	*Maghreb*	15.0	17.7
	*Eastern Europe*	6.4	2.9
	*Turkey*	3.3	3.2
	*Former-EU15*	11.4	9.0
	*South-East Asia*	1.7	0.8
**Education**	≤*9th grade*	19.7	25.9
	*High school*	36.5	46.7
	*College*	43.9	27.3
**Unemployed**		40.9	50.6
**Single mother**		5.5	6.1
**Delivery characteristics**			
**Cesarean section**		17.8	28.3
**Induction**		31.1	38.6
**Elective CS**		8.7	13.6
**Timing of induction or elective CS**	*<39 weeks*	35.0	41.0
	*39-40 weeks*	49.0	44.7
	≥*41 weeks*	15.9	14.3
**Gestational age**	*<37 weeks*	7.6	8.1
	*37-40 weeks*	81.9	80.5
	*≥41 weeks*	10.6	11.4
**Macrosomia**		6.8	11.6
**Emergency CS**		9.0	14.5
**Transfer to NICU**		10.9	15.4
**1 minute Apgar score < 7**		6.9	9.1
**Perinatal mortality**		0.6	0.7

*EU15*, Former 15-European Union member countries; *CS*, cesarean section; *NICU*, neonatal intensive care unit.

All differences were statistically significant with a *p*-value <0.001.

When considering all births, obese mothers had a 38% excess risk of admission to neonatal intensive care unit after adjustment for all characteristics (Table 2). The last model explains up to 23.1% of the excess neonatal admission, while adjustment for maternal factors including age, parity, height and weight gain only explained 1.1% of the excess risk (data not shown). Women in spontaneous or induced labor had also an excess risk of 45% and 34% respectively, while women with elective CS had a statistically non-significant excess risk of 18% after introducing medical characteristics in the multivariate model.

**Table 2:** Multivariate analysis of admission to neonatal intensive care unit in obese compared with non-obese women

**Obese vs. non obese-women**	**Crude OR (95%CI)**	**Adjusted OR (95%CI)**				**R**^**2**^
		**Model 1**	**Model 2**	**Model 3**	**Model 3 with hospital effect**	
All births (n = 36964)	1.49	1.54	1.43	1.38	1.38	23.1%
	(1.36-1.62)**	(1.41-1.69)**	(1.29-1.59)**	(1.23-1.56)**	(1.22-1.56)**	
Women in spontaneous labor (n = 21784)	1.54	1.61	1.49	1.45	1.45	27.4%
	(1.37-1.75)**	(1.42-1.82)**	(1.28-1.74)**	(1.22-1.72)**	(1.21-1.73)**	
Women with induction of labor (n = 11774)	1.43	1.48	1.34	1.31	1.34	14.2%
	(1.22-1.66)**	(1.27-1.73)**	(1.13-1.59)**	(1.08-1.59)**	(1.10-1.63)**	
Women with elective cesarean section (n = 3416)	1.34	1.45	1.30	1.17	1.18	26.8%
	(1.08-1.67)**	(1.16-1.81)**	(0.99-1.71)	(0.86-1.60)	(0.86-1.63)	

**p-value < 0.01; *vs*., versus; *OR*, odds ratio. *R*^*2*^, logistic regression pseudo-R^2^. N total = 36 959, 36 913 and 30133 in models 1, 2 and 3 respectively.

Model 1: Adjusted OR for maternal age, parity, maternal weight gain and height.

Model 2: Adjusted OR for model 1 and multiple birth, hypertension, diabetes, macrosomia, gestational age.

Model 3: Adjusted OR for model 2 and maternal origin, education, employment, cohabiting status.

When considering all births, obese mothers had a 31% excess risk of low Apgar score after adjustment for all characteristics (Table 3). Women in spontaneous or induced labor had also an excess risk of 26% and 38% respectively, while women with elective CS had an excess risk of 50% after introducing socio-economic characteristics in the multivariate model, though not statistically significant.

**Table 3:** Multivariate analysis of 1 minute Apgar score <7 in obese compared with non-obese women

**Obese vs. non obese-women**	**Crude OR (95%CI)**	**Adjusted OR (95%CI)**				**R**^**2**^
		**Model 1**	**Model 2**	**Model 3**	**Model 3 with hospital effect**	
All births (n = 38234)	1.35	1.42	1.36	1.30	1.31	4.0%
	(1.21-1.50)**	(1.27-1.58)**	(1.21-1.52)**	(1.14-1.48)**	(1.15-1.49)**	
Women in spontaneous labor (n = 22499)	1.38	1.45	1.35	1.25	1.26	4.0%
	(1.18-1.61)**	(1.24-1.69)**	(1.15-1.59)**	(1.03-1.51)*	(1.04-1.52)**	
Women with induction of labor (n = 12218)	1.23	1.29	1.31	1.35	1.38	4.1%
	(1.04-1.45)*	(1.09-1.53)**	(1.10-1.57)**	(1.11-1.66)**	(1.12-1.69)**	
Women with elective cesarean section (n = 3528)	1.76	1.83	1.75	1.50	NC	9.7%
	(1.25-2.47)**	(1.30-2.59)**	(1.21-2.54)**	(0.96-2.36)		

*p-value < 0.05; **p-value < 0.01; *vs*., versus; *OR*, odds ratio; *NC*, not calculable. *R*^*2*^, logistic regression pseudo-R^2^. N total = 38 230, 38 181 and 31 015 in models 1, 2 and 3 respectively.

Model 1: Adjusted OR for maternal age, parity, maternal weight gain and height.

Model 2: Adjusted OR for model 1 and multiple birth, hypertension, diabetes, macrosomia, gestational age.

Model 3: Adjusted OR for model 2 and maternal origin, education, employment, cohabiting status.

Obese mothers had a crude odds ratio of 1.19 (0.82-1.74) and an adjusted odds ratio with mixed-effects model of 1.36 (0.75-2.45) for perinatal mortality (Table 4). Women in spontaneous or induced labor and elective CS had a crude odds ratio for perinatal mortality of 1.86 (1.04-3.32), 0.85 (0.51-1.42), and 0.49 (0.06-3.90) respectively.

**Table 4:** Multivariate analysis of perinatal mortality in obese compared with non-obese women

**Obese vs. non obese-women**	**Crude OR (95%CI)**	**Adjusted OR (95%CI)**				**R**^**2**^
		**Model 1**	**Model 2**	**Model 3**	**Model 3 with hospital effect**	
All births (n = 38675)	1.19	1.28	1.29	1.34	1.36	21.5%
	(0.82-1.74)	(0.87-1.87)	(0.86-1.93)	(0.75-2.41)	(0.75-2.45)	
Women in spontaneous labor (n = 22732)	1.86	1.90	1.83	2.10	NC	17.7%
	(1.04-3.32)*	(1.06-3.42)*	(0.99-3.37)	(0.88-5.00)		
Women with induction of labor (n = 12363)	0.85	0.92	1.13	1.20	NC	36.6%
	(0.51-1.42)	(0.55-1.56)	(0.63-2.02)	(0.47-3.05)		
Women with elective cesarean section (n = 3591)	0.49	0.57	0.43	0.49	NC	26.0%
	(0.06-3.90)	(0.07-4.59)	(0.05-3.82)	(0.04-5.44)		

*p-value < 0.05; **p-value < 0.01; *vs*., versus; *OR*, odds ratio; *NC*, not calculable. *R*^*2*^, logistic regression pseudo-R^2^. N total = 38 670, 38 602 and 31 303 in models 1, 2 and 3 respectively.

Model 1: Adjusted OR for maternal age, parity, maternal weight gain and height.

Model 2: Adjusted OR for model 1 and multiple birth, hypertension, diabetes, macrosomia, gestational age.

Model 3: Adjusted OR for model 2 and maternal origin, education, employment, cohabiting status.

The likelihood ratio test of the mixed-effects model taking into account the hospital effect showed that there was a significant overall effect due to the hospital on the relation between obesity and neonatal admission and between obesity and Apgar score (*p*-value < 0.001) in all delivery subgroups, but the differences between the ordinary logistic results and the random-effect model were small.

The odds ratio with hospital effect for the Apgar score could not be calculated among women with elective CS because of the small number of women. Multivariate analyses with hospital effect could not be performed for perinatal mortality in the different delivery subgroups because of the small amount of perinatal deaths.

The analyses were repeated for pregnancies that ended up at 39 and 40 weeks of pregnancy. Results were similar with an adjusted OR (model 3 with hospital effect) of 1.47 (1.21-1.78) for admission to NICU and 1.28 (1.06-1.55) for 1 minute Apgar score inferior to 7 and with a crude odds ratio of 4.82 (1.87-12.46) for perinatal mortality.

## Discussion

The neonatal need for intensive care was increased by 38% in obese women in our population.

This finding could reflect low Apgar scores, as 1 minute Apgar score inferior to 7 was increased by 31% in obese mothers. Both women in spontaneous and induced labor had a significantly increased risk of neonatal admission and low Apgar score.

In agreement with our observations, the neonatal admission for intensive care was significantly increased in obese mothers in a recent meta-analysis including 4 studies [4], and a higher rate of admission to NICU in obese women has been previously observed in Europe, USA, Canada and Australia [7,11-19], even in term births [7]. However, most of these studies only adjusted for a small set of covariates such as age and parity or used weight categories rather than BMI and did not differentiate between induced and spontaneous labour [4,5]. Apgar scores were rarely reported [11,12,18,20,21].

The decreased relation observed after elective CS in our study should be discussed. It is important to keep in mind that women who undergo labor or elective CS both are heterogenous groups: they do not constitute a common cohort. First, women with elective CS could have had a particular maternal condition. Morbidity within the group of elective CS might be higher across BMI categories and may, thus, lead to comparable outcomes in obese and non-obese mothers [6]; second, it may also indicate that a reason for elective CS was identified and discussed with the mother, leading to better outcome. Circumstances of birth, as well as factors influencing clinical decision making, should be explored, as a hospital effect on the relation between obesity and all delivery outcomes was found; and third, the subgroup of women with elective CS was small, and it could limit the analysis.

The reason for this increased rate of neonatal complications in obese women is unknown but could be related to increased maternal pelvic soft tissue, as well as difficulty in estimating the fetal weight, and intrapartum complications such as inability to adequately monitor the fetus and contractions [4,20]. Although there is strong evidence for the relation between macrosomia and shoulder dystocia, the current evidence for an independent relation between maternal obesity and shoulder dystocia through the excess fat tissue in the birth canal is less clear [2,4,22,23]. Besides mechanical hypotheses, pregnancy is associated with wide-ranging cardiovascular changes through increased oxygen demand, and obesity-induced changes have profound effects on cardiac and vascular function [24]. Neonatal morbidity may be due to perturbed cardiovascular function in the mother and difficulties in hemodynamic adaptation during labor and delivery. Neonatal complications include hypoglycemia, jaundice, and respiratory distress [17,25], and in a small study investigating specifically children on the neonatal ward, children from obese mothers were characterized by a decreased need for oxygen administration and a shorter stay and further investigation is required [17,25].

Cesarean section can also be technically more difficult in obese women, and there is a higher risk of anaesthetic and postpartum complications compared with normal weight mothers. The decision for mode of delivery should therefore be taken only after careful consideration of the individual circumstances and in conjunction with the multidisciplinary team and the woman herself [20,26].

A limitation of this study is that we relied on self-reported prepregnancy BMI if the first antenatal was held late, and obese women may have underestimated their weight. However, considering that we found increased risk of adverse outcomes, the true risks would be even greater. Obese women may also refuse to be weighed or to mention their weight, but we evaluated the group of women with missing data on weight, height and ORs with regard to unfavourable outcomes were not increased compared with women with known BMI (data not shown). The item Diabetes in the birth certificates is defined as diabetes, either pre-existing or first recognized during pregnancy, regardless of the diagnostic criteria used, for ease of use. Universal screening by a glucose challenge test followed by an oral glucose tolerance test if the result exceeded 140 mg/dl was the usual procedure in 2009. The item Hypertension covers all forms of hypertension, either pre-existing or first recognized during pregnancy, and is defined in the birth certificates as a systolic blood pressure of at least 140 mmHg and/or a diastolic blood pressure of at least 90 mmHg. Several techniques and definitions may have been used across the country, and we cannot preclude that this could affect our results.

It is also of note that one cannot reliably determine from birth certificates whether the vaginal delivery was a planned and monitored event, or whether it was a precipitous delivery of a high-risk pregnancy [27]. The analyses were therefore repeated in pregnancies after 39 weeks to preclude an effect of early induction of labor or elective CS before 39 weeks. Another limitation of this study is that the numbers were not large enough to assess the link between obesity and perinatal death.

The strength of our study is that it is population-based with a low rate of missing data and a large set of covariates. Very few studies reporting delivery outcomes in obese mothers have utilised statistical methods to adjust for socio-economic and hospital characteristics. This is also the first study to assess the relation between obesity and infant outcomes in women according to type of labor and delivery mode, although a recent study has highlighted the importance of delivery mode and induction in evaluating neonatal mortality rates in obese women [6]. Despite the great number of studies focused on pregnancy complications in obese mothers, few data are available on neonatal care and the extra-cost induced by obesity and further research is needed [4,5,17].

## Conclusion

Admission to neonatal intensive care unit and low Apgar scores are more frequent for infants of obese mothers, both after spontaneous and induced labor. While it is of crucial importance to prevent obesity in pregnancy, further assessment of timing of delivery and delivery mode in obese women is necessary as well as the identification of mediators of the excess neonatal risk.

## Abbreviations

CS: Cesarean section; BMI: Body mass index; NICU: Neonatal intensive care unit; OR: Odds ratio; 95%CI: 95% confidence interval.

## Competing interests

The authors declare that they have no competing interests.

## Authors’ contributions

AFM participated in gathering and correcting the database and drafted the manuscript. PB participated in the design of the study and helped to draft the manuscript. MD participated in gathering and correcting the database and revising the manuscript. YE participated in the design of the study and participated in gathering and correcting the database and helped to draft the manuscript. All authors read and approved the final manuscript.

## Pre-publication history

The pre-publication history for this paper can be accessed here:

http://www.biomedcentral.com/1471-2393/13/36/prepub
